# Acute myocarditis and low melatonin: unraveling a potential link

**DOI:** 10.3389/fcvm.2025.1580934

**Published:** 2026-01-13

**Authors:** Qun Chen, Keyi Wang, Xiu-zhen Long, Jie Sun, Ying-ran Li, Wen-yuan Zhang

**Affiliations:** 1 Department of Pharmacy, Zhongshan City People’s Hospital, Zhongshan, China; 2 School of Pharmaceutical Sciences, Zunyi Medical University, Zunyi, China; 3 Imaging Center, Zhongshan City People’s Hospital, Zhongshan, China; 4 Department of Cardiology, Zhongshan City People’s Hospital, Zhongshan, China

**Keywords:** 6-sulphatoxymelatonin, cardioprotection, markers, melatonin, myocarditis

## Abstract

**Background:**

Acute myocarditis is one of the common causes of sudden cardiac death among young adults. While melatonin has been recognized for its cardioprotective properties, the specific relationship between melatonin and acute myocarditis in humans is not well established.

**Methods:**

We collected morning urine samples from 21 patients diagnosed with acute myocarditis and 21 healthy controls to measure the levels of 6-sulfatoxymelatonin (aMT6s), a biomarker of nocturnal melatonin secretion, using an ELISA assay.

**Results:**

The mean age of the control group was 31.05 ± 9.75 years, and the acute myocarditis group had a mean age of 30.71 ± 10.11 years. Both groups were evenly divided by gender, with 15 males and 6 females in each. Acute myocarditis patients exhibited significantly lower aMT6s levels (50.57 ± 36.39 ng/mL) compared with healthy volunteers (80.36 ± 48.92 ng/mL; *P* = 0.031). Similarly, the aMT6s-to-creatinine ratio was reduced in patients (106.95 ± 73.45 ng/mg cr) vs. controls (159.73 ± 92.96 ng/mg cr; *P* = 0.048).

**Conclusion:**

Lower melatonin levels, measured via urinary aMT6s concentrations in acute myocarditis patients, suggest a link to the disease process.

## 1 Introduction

Acute myocarditis, characterized by inflammatory myocardial damage, is a common cause of sudden cardiac death, particularly among young population (1). While viral infections are the primary triggers of myocardit, it can also be precipitated by bacterial, protozoal, or fungal infections, as well as exposure to various toxic substances, medications, and systemic immune-mediated disorders (2). In most cases, treatment is primarily supportive, focusing on managing complications such as heart failure or arrhythmias (3). Given the diverse clinical manifestations and the multitude of etiological factors associated with myocarditis, it is crucial to identify potential biomarkers to advance our understanding of myocarditis.

Melatonin, a hormone that regulates biological rhythms, has been observed to possess cardioprotective effects. As comprehensively reviewed by Favero et al. (4), melatonin exerts cardiac protection through multiple mechanisms, including direct free radical scavenging, mitochondrial function preservation, and suppression of inflammatory cascades. Preclinical animal studies provide mechanistic insights for these effects. For example, melatonin has been shown to mitigate cardiac injury in a mouse model of myocardial infarction (MI) (5), as well as to preserve cardiac function and slow the progression of heart failure (6). In the clinical setting, patients with MI or dilated cardiomyopathy (DCM) exhibit lower circulating levels of endogenous melatonin compared to control subjects, with these reduced levels correlating with the severity of myocardial damage and cardiac output (7). Additionally, a placebo-controlled clinical trial has demonstrated that the administration of melatonin to patients with STEMI who presented early after symptom onset was associated with a significant reduction in infarct size following primary percutaneous coronary intervention (pPCI) (8).

Despite these findings highlighting melatonin's role in cardiac injury, its specific involvement in acute myocarditis remains poorly defined. Preclinical evidence confirms melatonin's protective role against viral myocarditis [e.g., CVB3-induced myocarditis (9, 10)] and sepsis-driven myocarditis (11). However, clinical studies evaluating endogenous melatonin levels in patients with acute myocarditis are currently lacking. Notably, our metabolomic study in acute myocarditis patients revealed increased urinary N-formylkynurenine (12), suggesting a possible inflammatory-driven shift in tryptophan metabolism toward the kynurenine pathway. We hypothesize that upregulation of the kynurenine pathway occurs at the expense of melatonin synthesis, leading to reduced endogenous melatonin levels.

## 2 Methods

A total of 21 patients with acute myocarditis were enrolled from the Department of Cardiology at Zhongshan City People's Hospital between October 2022 and December 2023. The diagnosis was confirmed by cardiac MRI based on criteria established by the Circulation Journal (13). Simultaneously, 21 age- and sex-matched healthy controls were recruited as volunteers. The exclusion criteria for both groups included: intake of melatonin supplements or sedative-hypnotic medications within the past month, engagement in shift work, and, for patients only, the presence of coronary artery lesions determined by coronary angiography or coronary CTA. Prior to participation, each individual provided written informed consent, and the study was approved by the Ethics Committee of Zhongshan City People's Hospital, adhering to the Declaration of Helsinki revised in 2024 and laid down in 1964.

We systematically collected comprehensive patient information, including key clinical indicators such as N-terminal pro-B-type natriuretic peptide (NT-proBNP), troponin T (TnT), and creatine kinase isoenzyme MB (CK-MB). Acknowledging their potential as confounders, we also collected specific factors known to influence melatonin secretion: inflammatory biomarkers [e.g., high-sensitivity C-reactive protein (Hs-CRP)], medication use (e.g., beta-blockers, corticosteroids), and overall sleep quality using the Pittsburgh Sleep Quality Index (PSQI) (14). Healthy control subjects who met the inclusion criteria were screened using a comprehensive health questionnaire to exclude underlying illnesses and disorders, and their sleep quality was similarly assessed using the PSQI. Nevertheless, we recognize the limitation that several other potential confounders affecting melatonin secretion, such as detailed nocturnal light exposure and objective measures of acute stress, were not systematically recorded in this study.

We collected first-morning, mid-stream urine samples from all participants after an overnight fast, with samples from acute myocarditis patients were obtained within 48 h of diagnosis. To preserve sample integrity, we processed all samples within 2 h of collection. This involved centrifugation at 1,000 × g for 15 min, aliquoting of the supernatant, and immediate transfer to a dedicated −80 ℃ freezer for stable storage until analysis.

We used the enzyme-linked immunosorbent assay (ELISA, Cat No:RE54031, IBL International GmbH) to measure the concentration of 6-sulphatoxymelatonin (aMT6s) in the two groups to assess melatonin secretion levels. Melatonin is primarily metabolized in the liver to aMT6s and excreted in the urine. Hence, the measurement of aMT6s in the first morning void is a well-established, non-invasive method for estimating integrated nocturnal melatonin secretion (15, 16). This approach is subject to certain limitations, including inherent day-to-day physiological variability and potential confounding effects from individual differences in renal function or urine concentration. To mitigate the influence of variations in urine dilution, aMT6s concentrations were normalized to urinary creatinine levels (expressed as aMT6s/Cr ratio). Urinary creatinine was quantified using a commercially available ELISA kit (Cat No: JM-6769H1, Jiangsu Jingmei Biological Technology Co., Ltd.). The robustness and analytical accuracy of both ELISA methods employed have been previously established in the literature (17, 18).

### 2.1 Statistical analysis

SPSS 29.0 software was used for statistical analysis. The receiver operating characteristic (ROC) curve was generated using the ROC Curve procedure. The Shapiro–Wilk test was used to assess the normality of the data. Normally distributed data are expressed as mean (M) ± standard deviation (SD) and were compared using the two-tailed t-test. Non-normally distributed data were expressed as the median and interquartile range (IQR). *P* < 0.05 was considered significant.

## 3 Results

The mean age of the control group was 31.05 ± 9.75 years, while the myocarditis group had a mean age of 30.71 ± 10.11 years. Both groups had the same number of male and female participants, with 15 males and 6 females in each. In the cohort of patients with acute myocarditis, a substantial majority, specifically 85.7%, reported symptoms of chest distress and/or pain. Moreover, a noteworthy 90.4% of these patients exhibited signs of infection prior to hospital admission, manifesting as fever, cough, rhinorrhea, or diarrhea. According to the proposed stages of myocarditis from the ACC Expert Consensus Decision Pathway on Strategies and Criteria for the Diagnosis and Management of Myocarditis (19), 19 patients were classified as Stage C (symptomatic myocarditis) and 2 patients as Stage D (advanced myocarditis). Clinical assessment confirmed myocardial injury in these patients. Electrocardiogram identified ST-segment elevation in 10 patients (47.6%), suggesting acute myocardial involvement. Echocardiography revealed a median left ventricular ejection fraction of 62%, with 4 patients (19.0%) exhibiting moderate to severe cardiac dysfunction (LVEF < 50%), and laboratory tests demonstrated significantly elevated biomarkers of myocardial necrosis, including a median NT-proBNP level of 277 pg/mL, with an interquartile range (IQR) extending from 125 to 1,946 pg/mL. TnT levels had a median value of 469 ng/L, with an IQR of 267 to 1,578 ng/L. CK-MB levels showed a median of 27 U/L, with an IQR from 13 to 58 U/L. CMR results showed that the median myocardial T1 value was 1,119 ms (IQR: 1,078–1,167 ms). The median T2 value was 60 ms (IQR: 54.5–62.5 ms). During hospitalization, all patients received standard supportive treatment, including rest and cardioprotective medications (such as multivitamins, trimetazidine hydrochloride, etc.). Regarding medication, 11 patients (52.4%) used beta-blockers, and 4 patients (19.0%) used corticosteroids (Table 1). The median length of hospital stay was 7 days (IQR: 6–9.5). No deaths occurred during the course of the disease.

**Table 1:** Comparison of baseline characteristics and aMT6s concentration between acute myocarditis patients and healthy controls.

Variable	Myocarditis (*N* = 21)	Control (*N* = 21)	*P*
Age, Mean (SD), years	30.71 (10.11)	31.05 (9.75)	ns
Male, *n* (%)	15 (71.4)	15 (71.4)	ns
Symptom			
– Chest distress and/or pain (%)	85.7%	–	–
Signs of infection before hospital admission (%)	90.4%	–	–
Cardiac Function Indicators			
LVEF[%, Median (Q1,Q3)]	62 (56.5, 67.5)	–	–
ST-segment elevation appears, *n* (%)	10 (47.6)	–	–
Hs-CRP[mg/L, Median (Q1,Q3)]	49.5 (9.2, 49.5)	–	–
NT-proBNP [pg/mL,M(Q1,Q3)]	277 (125, 1,946)	–	–
TnT[ng/L, M(Q1,Q3)]	469 (267, 1,578)	–	–
CK-MB[U/L, M(Q1,Q3)]	27 (13, 58)	–	–
Medication usage			
Beta-blockers, n(%)	11 (52.4)	–	–
Corticosteroids, n(%)	4 (19.0)	–	–
Melatonin Levels			
aMT6s, Mean (SD) (ng/mL)	50.57 (36.39)	80.36 (48.92)	*P* < 0.05
aMT6s/Cr (ng/mg cr)	106.95 (73.45)	159.73 (92.96)	*P* < 0.05

LVEF, left ventricular ejection fraction; Hs-CRP, high-sensitivity C-reactive protein; TnT, troponin T; CK-MB, creatine kinase-MB; aMT6s, 6-sulfatoxymelatonin; SD, standard deviation.

**Figure 1:**
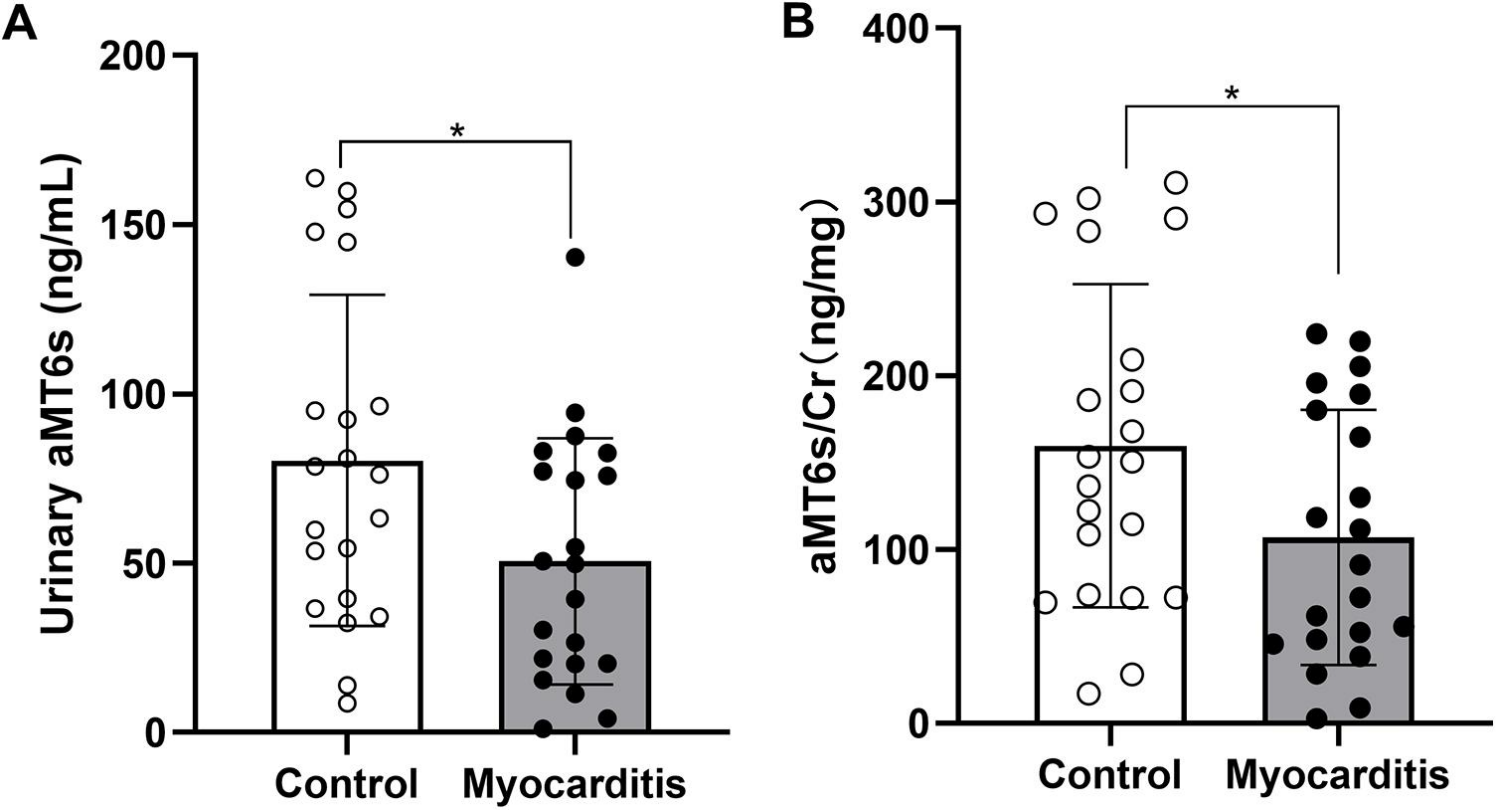
Comparison of aMT6s concentration and normalized to urinary creatinine levels (expressed as aMT6s/Cr ratio) between the acute myocarditis group and the healthy control group. Data are presented as mean ± SD. **(A)** Urinary aMT6s concentration was significantly lower in the acute myocarditis group compared with controls (*P* = 0.031, *t*-test; Cohen's d = 0.691, 95% CI: 0.064–1.310). **(B)** The aMT6s-to-creatinine ratio was also reduced in myocarditis patients (*P* = 0.048, *t*-test; Cohen's d = 0.630, 95% CI: 0.006–1.247). *p < 0.05 compared to the Control group.

**Figure 2:**
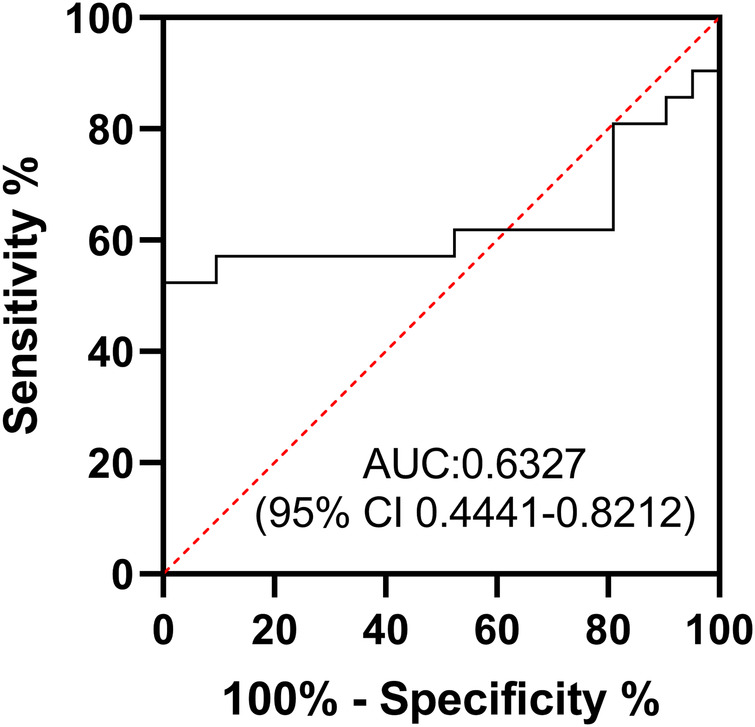
Receiver operating characteristic (ROC) curve of aMT6s/Cr. The diagnostic performance of morning urinary creatinine-corrected aMT6s for acute myocarditis (AUC = 0.63, 95% CI: 0.4441–0.8212).

In addition, we compared the sleep quality of patients with myocarditis to healthy controls. Overall sleep quality was poorer in patients with myocarditis, with significantly higher PSQI total scores compared to the control group (M ± SD: 6.19 ± 2.38 vs. 4.33 ± 2.82, *P* = 0.026, 95%CI: 0.084–1.333, Supplementary Table 1). Normality of distribution for aMT6s and aMT6s/Cr was assessed using the Shapiro–Wilk test, which confirmed that these data in both groups did not significantly deviate from normality (both *P* > 0.05). Acute myocarditis patients showed markedly lower aMT6s levels (M ± SD: 50.57 ± 36.39 ng/mL; 95%CI: 34.0–67.1; Median: 49.8 ng/mL) compared with healthy volunteers (M ± SD: 80.36 ± 48.92 ng/mL; 95%CI: 58.1–102.6; Median: 76.3 ng/mL), with the aMT6s-to-creatinine ratio also reduced in patients (M ± SD: 106.95 ± 73.45 ng/mg cr; 95%CI: 73.5–140.4; Median: 91.3 ng/mg cr) compared to controls (M ± SD: 159.73 ± 92.96 ng/mg cr; 95%CI: 117.4–202.1; Median: 150.6 ng/mg cr) (Table 1; Figure 1). Both uncorrected aMT6s (*P* = 0.031) and creatinine-normalized values (*P* = 0.048) remained significantly lower in myocarditis patients (two-tailed *t*-tests), demonstrating moderate effect sizes (Cohen's d = −0.691, 95% CI: −1.310 to −0.064 for aMT6s; d = −0.630, 95% CI: −1.247 to −0.006 for aMT6s/Cr). Receiver operating characteristic curve analysis indicated that the morning urinary aMT6s-to-creatinine ratio yielded an area under the curve (AUC) of 0.63 (95% CI: 0.4441–0.8212) for distinguishing patients with acute myocarditis from healthy controls (Figure 2).

## 4 Discussion

Melatonin possesses a range of beneficial properties, such as the ability to resynchronize biological rhythms, act as an antioxidant, reduce inflammation, dampen excitatory signals, promote sleep, and modulate the immune system, which have been shown to be advantageous in cardiovascular disease management (20). In our study, we observed a significant decrease in nocturnal melatonin secretion levels among patients with acute myocarditis when compared to healthy controls. The magnitude of this decrease was approximately 33%, which is less pronounced than the 57% reduction previously reported in patients with acute myocardial infarction (15). The etiology of suppressed melatonin in our cohort is likely multifactorial. One intriguing hypothesis is that inflammation diverts tryptophan metabolism away from melatonin synthesis. Preclinical evidence shows that viral myocarditis models exhibit activated Indoleamine 2,3-dioxygenase (IDO), which shifts tryptophan toward the kynurenine pathways; notably, inhibiting IDO reduces mortality and viral load by preserving antiviral immunity (21). Clinically, Mendelian randomization studies confirm that kynurenine pathway activation elevates myocarditis risk (22) and that urinary N-formylkynurenine levels are specifically elevated in myocarditis patients (12), suggesting potential competition with melatonin synthesis.

The observed reduction in melatonin may also reflect a general acute stress response rather than a myocarditis-specific phenomenon. Viral infections themselves can directly influence melatonin levels; for instance, one study reported approximately 20% lower serum melatonin levels in patients with mild-to-moderate COVID-19 compared to a non-COVID-19 infection control group (23). Systemic viral symptoms—such as fever and sleep disruption—may further disturb circadian rhythms and suppress melatonin production. In line with this, we found that patients with acute myocarditis exhibited poorer sleep quality according to the PSQI relative to healthy controls. Iatrogenic factors also likely contributed: four patients received corticosteroid therapy, and eleven received beta-blockers, both of which are associated with reduced melatonin secretion (15).

The diagnostic value of urinary aMT6s for myocarditis appears limited. Our analysis showed that morning urinary creatinine-corrected aMT6s had an ROC-AUC of only 0.63, indicating weak diagnostic power. However, its potential role in prognostic assessment, particularly in severe myocarditis, warrants further investigation in future studies. Recent clinical evidence has established the disruption of circadian melatonin rhythm as a prognostic biomarker for adverse outcomes in critically ill patients (24). Furthermore, preclinical studies strongly support the therapeutic benefits of melatonin in myocarditis. For instance, Ouyang et al. (10) discovered that melatonin mitigates virus-induced endoplasmic reticulum (ER) stress and preserves mitochondrial function, thereby alleviating viral myocarditis. Moreover, melatonin exhibits a potent therapeutic impact on acute viral myocarditis, which is linked to its capacity to regulate autophagy and inhibit apoptosis within the heart (9). In another study, melatonin was found to reduce cardiac inflammation by decreasing the levels of IL-1*α*, IL-1β, IL-16, and MCP-1 messenger RNA (mRNA) in a myocarditis model (11).

Lower melatonin levels, measured via urinary aMT6s concentrations in acute myocarditis patients, are associated with the disease state, warranting further investigation to define its precise role.

## 5 Study limitations

We acknowledge four key limitations: (1) The low incidence of myocarditis resulted in a modest sample size, potentially limiting generalizability; (2) Our cross-sectional design precludes causal inference between acute myocarditis and melatonin levels and lacks longitudinal follow-up to assess the long-term dynamics of melatonin; (3) Critical confounding factors were unmeasured: nocturnal light exposure and acute stress; (4) The absence of severity stratification limits our ability to explore potential associations between melatonin levels and the clinical severity of myocarditis. Despite these limitations, our findings provide foundational insights for future research on melatonin's role in acute myocarditis.

## Data Availability

The original contributions presented in the study are included in the article/Supplementary Material, further inquiries can be directed to the corresponding author.
