# Supplementary material for: Acute myocarditis and low melatonin: unraveling a potential link
Source: Front Cardiovasc Med. 2026 Jan 13;12:1580934. doi: 10.3389/fcvm.2025.1580934 (PMC12835375; doi:10.3389/fcvm.2025.1580934)
Supplement: Supplementary file 1 [file Datasheet1.docx]

Supplementary materials

Pittsburgh Sleep Quality Index (PSQI)

The Pittsburgh Sleep Quality Index (PSQI) was employed to assess the participants' overall sleep quality over the preceding month (1). It is one of the most widely used clinical instruments for evaluating sleep quality. The PSQI has been validated in the Chinese population, with studies confirming its satisfactory reliability and validity, making it suitable for sleep medicine research in China. The index consists of 5 other-rated items and 19 self-rated items, which are categorized into seven components for secondary statistical analysis: subjective sleep quality, sleep latency, sleep duration, habitual sleep efficiency, sleep disturbances, use of sleep medication, and daytime dysfunction. Each component is scored on a scale of 0 to 3. The global PSQI score is the sum of these component scores, with a higher total score indicating poorer sleep quality. A global score ≤ 7 is defined as normal sleep, whereas a score > 7 indicates the presence of sleep disturbance.

Supplementary Table S1 Comparison of overall sleep quality between patients with myocarditis and healthy individuals

|  | Myocarditis (N=21) | Control (N=21) | t-value | *P* |
| --- | --- | --- | --- | --- |
| PSQI Total Score | 6.19±2.38 | 4.33±2.82 | -2.431 | **0.026** |
| PSQI＞7（%） | 7（33.33%） | 3（14.29%） | - | - |
| subjective sleep quality | 1.29±0.664 | 0.86±0.573 | -2.140 | **0.032** |
| bedtime | 0.95±0.973 | 0.76±0.831 | -0.581 | 0.561 |
| sleep time | 1.10±0.944 | 0.95±0.669 | -0.314 | 0.754 |
| sleep efficiency | 0.14±0.478 | 0.19±0.512 | -0.447 | 0.655 |
| sleep disturbance | 0.90±0.436 | 0.71±0.561 | -1.276 | 0.202 |
| Use of hypnotic drugs | 0.00±0.000 | 0.00±0.000 | 0.000 | 1.000 |
| daytime dysfunction | 1.81±0.873 | 1.10±1.044 | -2.257 | **0.024** |

Data are presented as mean ± SD. Group difference was analyzed using an unpaired two-tailed Student's t-test.

1. Mollayeva T, Thurairajah P, Burton K, Mollayeva S, Shapiro CM, Colantonio A. The Pittsburgh Sleep Quality Index as a Screening Tool for Sleep Dysfunction in Clinical and Non-Clinical Samples: A Systematic Review and Meta-Analysis. *Sleep medicine reviews* (2016) 25:52-73. Epub 2015/07/15. doi: 10.1016/j.smrv.2015.01.009.
